# Structure and Properties of Foam Concrete and Fiber-Reinforced Foam Concrete Produced Using a Complex Nanomodifier Based on Industrial Waste

**DOI:** 10.3390/ma19081517

**Published:** 2026-04-10

**Authors:** Diana M. Shakhalieva, Evgenii M. Shcherban’, Sergey A. Stel’makh, Levon R. Mailyan, Andrei Chernil’nik, Natalya Shcherban’, Alexandr Evtushenko, Alexey N. Beskopylny

**Affiliations:** 1 Department of Design, Don State Technical University, 344003 Rostov-on-Don, Russia; delshaeva@donstu.ru; 2 Department of Engineering Geometry and Computer Graphics, Don State Technical University, 344003 Rostov-on-Don, Russia; etsherban@donstu.ru; 3 Department of Unique Buildings and Construction Engineering, Don State Technical University, 344003 Rostov-on-Don, Russia; sstelmah@donstu.ru (S.A.S.); lrm@aaanet.ru (L.R.M.); achernilnik@donstu.ru (A.C.); ndocenko@donstu.ru (N.S.); aevtushenko@donstu.ru (A.E.); 4 Department of Transport Systems, Faculty of Roads and Transport Systems, Don State Technical University, 344003 Rostov-on-Don, Russia

**Keywords:** foam concrete, fiber-reinforced foam concrete, complex nanomodifying additive, polypropylene fiber, nanoadditive, compressive strength

## Abstract

Foam concrete and fiber-reinforced foam concrete are promising building materials for sustainable and energy-efficient construction. Improving the environmental performance of cellular composites through the use of industrial waste and additives based on them is highly relevant. This study intends to create a novel complex nanomodifying additive (CNA) from industrial waste and nanomaterials, alongside new eco-friendly foam concrete (FC) and fiber-reinforced foam concrete (FFC) mixes incorporating CNA and polypropylene fiber (PF). Experimental studies yielded the optimal CNA formulation and described a method for its preparation. The test results indicate that FC’s properties are enhanced by CNA. The properties were best in the FC that was modified with 10% CNA. The FC control composition was surpassed by a 25.5% increase in compressive strength and a 23.1% increase in flexural strength, with a 9.5% reduction in thermal conductivity. Dispersed PF reinforcement also positively impacts the properties of FFCs with CNA, and the combined modification of 10% CNA and 1.2% PF provides maximum increases in compressive and flexural strength, amounting to 43.1% and 102.2%, respectively, and a 16.9% reduction in thermal conductivity. A microstructural analysis of the cellular composites confirms the feasibility of the tested formulation solutions. The FFCs, when modified by CNA and PF, display a homogeneous cellular structure, and the interpore zones contain multiple clusters of calcium silicate hydrate. Using CNA in the production of FC and FFCs will reduce cement consumption and improve their environmental friendliness.

## 1. Introduction

Energy conservation and carbon dioxide emission reduction are currently among the most pressing issues in the global community, attracting researchers from various industries, including the construction industry. Current construction trends are aimed at constructing environmentally friendly and energy-efficient buildings with comfortable conditions [1,2,3,4]. In this regard, foam concrete is seen as a beneficial building composite due to its thermal insulation properties and low cost [5,6]. The main raw material component of foam concrete is cement. Cement production is accompanied by large CO_2_ emissions, which account for approximately 8% of global emissions. Using fly ash in the manufacture of FC as a binder is considered an effective formulation technique that allows for savings in cement consumption and further improves the composite’s properties [7,8,9]. The inclusion of a mixture of fly ash (FA) and lime slurry as a mineral additive, replacing up to 80% of the cement, makes it possible “to obtain FC with characteristics that meet regulatory requirements” [10]. The addition of up to 30% FA and phase-changing materials to the composition of foam concrete increases its energy efficiency [11]. Foam concrete’s strength and water resistance are enhanced by carbonized fly ash, used optimally up to 40% [12]. The positive effects of including FA in the composition of foam concrete have also been recorded in other studies [13,14,15,16]. Microsilica is a common pozzolanic material, which is used in cement binder technology as a mineral additive and actively enters into hydration reactions. Concretes modified with waste incineration ash and microsilica (MS) demonstrate increased strength [17]. MS in self-healing concrete accelerates healing and completely heals “cracks with a width of 0.55 mm” [18]. Self-compacting concrete with MS up to 15% demonstrate improved mechanical properties, reduced water absorption, and increased resistance to the effects of sulfate and chloride environments [19]. The addition of wollastonite and 6% MS to self-compacting concrete significantly improves its flexural and tensile strength [20]. MS particles improve the strength of interfacial adhesion, reduce degradation, and increase durability under freeze–thaw cycles [21]. Other studies have similarly reported better mechanical properties and durability in composites after MS modification [22,23,24]. Nanomaterials are actively used in various industries, including the manufacture of building materials. Nanoparticles have unique properties that make it possible to optimize and improve the properties of cement composites [25,26]. Nanoparticles can act as microfillers, i.e., fill pores and generally optimize the nature of the pore structure of the composite. In addition, nanoparticles actively enter into chemical reactions and enhance interfacial adhesion [27,28]. Aluminum oxide nanoparticles (NA) are one of the types of nanomaterials that are used in the technology of cement composites to improve their properties [29]. Including 0.18% NA in the composition of FC has a stabilizing effect on the foam, and, as a result, a composite with a homogeneous fine-pored structure and improved properties is formed [30]. A modification of 1.0% NA improves the adhesion of cement composites to steel rods [31]. The compressive and tensile strength of self-compacting composites containing NA increased by 59.1% and 119%, respectively [32]. Moreover, the inclusion of NA in an amount of up to 3% improves the microstructure of concrete, making it denser. At the same time, the compressive strength increased by 24.6% [33]. Adding NA to 3D-printed concrete enhances printability and the final composite’s physical and mechanical characteristics [34]. NA particles accelerate hydration reactions, due to which the early strength of concrete increases to 13.8%, and the ability of concrete to penetrate chloride ions also increases [35]. Improvements in the properties of concrete composites with the inclusion of the NA nanomodifying additive were noted in the following studies [36,37,38]. The dispersed reinforcement of composites with polypropylene fiber is a common formulation technique and, with the optimal selection of the reinforcement percentage, can significantly improve their strength properties [39,40]. For example, the addition of “rubber particles and polypropylene fibers (PFs)” to foam concrete increases the compressive strength by up to 50% [41]. Twisted polypropylene fibers in amounts of up to 2% improve the mechanical properties of FC and increase its deformability [42]. Polymer foam mixtures for 3D printing with PF also demonstrate high performance properties [43]. Dispersed fiber reinforcement improves the dynamic properties of foam concrete. The presence of dispersed fibers in foam concrete composites ensures the redistribution of loads that arise under various mechanical and dynamic influences. For example, the inclusion of PF can increase the crack resistance of foam concrete pore walls under impact loads [44]. The addition of rubber particles and PF can increase the dynamic strength of foam concrete and the dynamic modulus of elasticity [41,45]. The inclusion of various types of fibers in the composition of foam concrete made from iron ore waste also improves the composite’s resistance to dynamic loads and, depending on the engineering requirements, can ensure the desired balance of strength and impact toughness [46]. Studies [47,48,49,50,51] showcase the beneficial impact of scattered PF reinforcement on foam concrete’s characteristics.

Thus, based on this short review, it can be noted that the modification of FC with industrial waste, including microsilica and fly ash, the incorporation of NA nanomaterials, and dispersed reinforcement, is a common formulation technique that allows for the production of environmentally friendly and cost-effective lightweight concrete with improved performance properties, which is confirmed by numerous scientific studies [52,53]. However, the widespread use of industrial-waste FA and MS is always limited by the unstable chemical composition of the waste and the degree of its readiness for introduction into the concrete mix. FA and MS often contain contaminants and foreign impurities, and have varying moisture contents, and large agglomerated accumulations. Before use, these wastes must undergo the appropriate preparation stages, namely, cleaning, drying, and sifting to remove agglomerated lumpy accumulations. In most scientific papers, the authors develop formulations for effective composites using specific types of waste, but do not address the issue of creating a complex modifying additive based on industrial waste and nanomaterials as a separate product for mass application and commercial sale [54,55]. This fact is a significant limitation and requires the search for appropriate solutions. This study is aimed at developing a formulation and technology for the preparation of a complex nanomodifying additive for cellular cement composites, and evaluating its effectiveness in foam concrete and fiber-reinforced foam concrete. The research innovation of the work aims to develop a new complex nano-modifying additive based on industrial waste and new environmentally friendly foam concrete and fiber-reinforced foam concrete compositions modified with such an additive and reinforced with polypropylene fiber, and the revealing of new dependencies of the properties and structure of the cellular composites on the composition and technological parameters. The aim of this work is to produce a final product in the form of a complex nanomodifying additive for cellular concrete and to develop environmentally friendly and energy-efficient structural and thermal insulation foam concrete (FC) and fiber-reinforced foam concrete (FFC) for green engineering.

## 2. Materials and Methods

### 2.1. Materials

The study consisted of the following stages:

– Studying the morphology of FA, MS, and NA particles, determining their chemical and phase composition using SEM and XRD methods, and determining the physical and mechanical properties of the raw materials;
– Developing a complex nanomodifying additive and evaluating its properties;
– Producing experimental foam concrete and fiber-reinforced foam concrete samples with varying dosages of the complex nanomodifying additive (CNA) and poly-propylene fiber (PF);
– Determining the workability parameters of foam concrete mixtures and the density, compressive strength, flexural strength, and thermal conductivity coefficients of hardened FC and FFC;
– Studying the structural features of FC and FFC using SEM methods;
– Analysis of experimental results and selection of the most optimal parameters for CNA modification and PF dispersion reinforcement parameters;
– Evaluation of the effectiveness of the developed FC and FFC formulations and comparison of structural quality factors.

The primary binder for making the experimental foam concrete (FC) samples was Portland cement CEM I 42.5 N (PC) sourced from CEMROS, Stary Oskol, Russia.

Table 1 details the fundamental properties of Portland cement.

Fine aggregate was sourced from quartz sand (QS) found in Nedra, Samarskoye village, Russia. Table 2 outlines the essential qualities of the sand.

**Figure 1:**
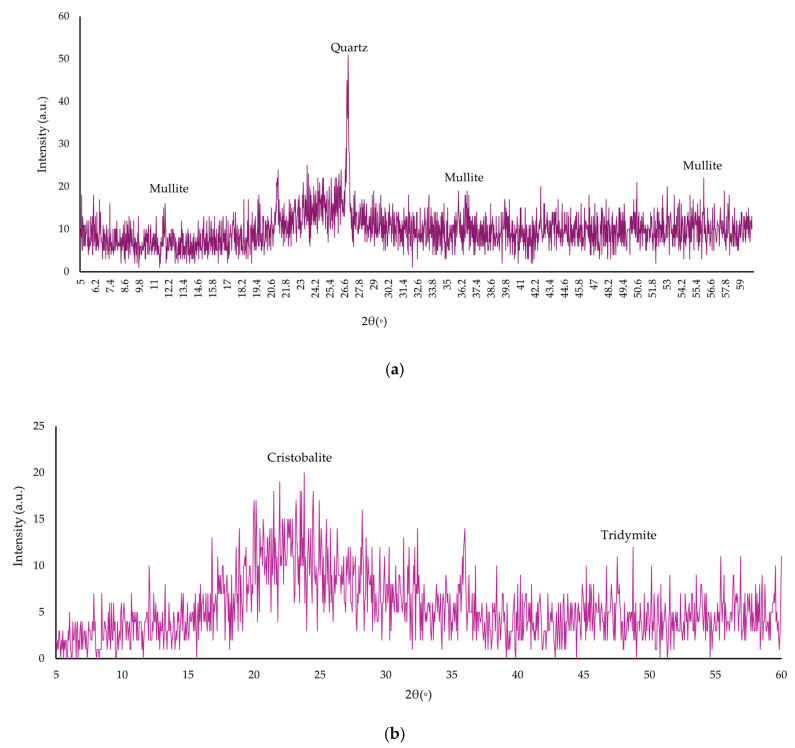
X-ray diffraction pattern: (**a**) FA; and (**b**) MS.

**Figure 2:**
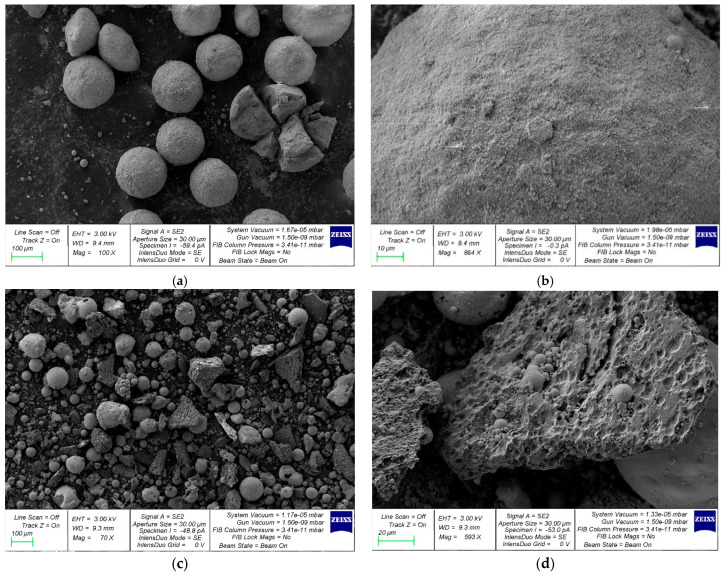
SEM image: (**a**) FA—scale 100 µm; (**b**) FA—scale 10 µm; (**c**) MS—scale 100 µm; and (**d**) MS—scale 20 µm.

The basis for the preparation of a complex nanomodifier based on industrial waste was fly ash (FA) (Novocherkassk State District Power Plant, Novocherkassk, Russia) and microsilica (MS) (NLMK, Lipetsk, Russia). Chemical composition of MS was as follows: SiO_2_—92.1%; Al_2_O_3_—0.66%; Fe_2_O_3_—0.85%; CaO—1.5%; MgO—1.03%; Na_2_O—0.61%; K_2_O—1.23%; C—0.94%; S—0.27%; and LOI—0.81%. Chemical composition of FA was as follows: SiO_2_—40.92%; TiO_2_—0.87%; Al_2_O_3_—21.9%; Fe_2_O_3_—9.38%; CaO—0.82%; MgO—1.68%; MnO—0.36%; K_2_O—5.25%; Na_2_O—0.9%; and LOI—17.92%. The bulk density for FA is 932 kg/m^3^ and for MS 152 kg/m^3^. The results of XRD and SEM analyzes of FA and MS are presented in Figure 1 and Figure 2, respectively.

The FA X-ray diffraction pattern (Figure 1a) reveals amorphous halos and reflections of quartz and mulit. A predominantly amorphous phase is shown by the MS X-ray diffraction pattern (Figure 1b), alongside reflections from tridymite and cristobalite. The mineral components are highly reactive due to the significant amorphous phase content. Calcium silicate hydrous (CSH) gels are readily formed when amorphous phases interact with Ca(OH)_2_ and water.

FA and MS particles are spherical. MS also exhibits particles of irregular geometric shapes. Observed are agglomerated clusters of MS particles.

**Figure 3:**
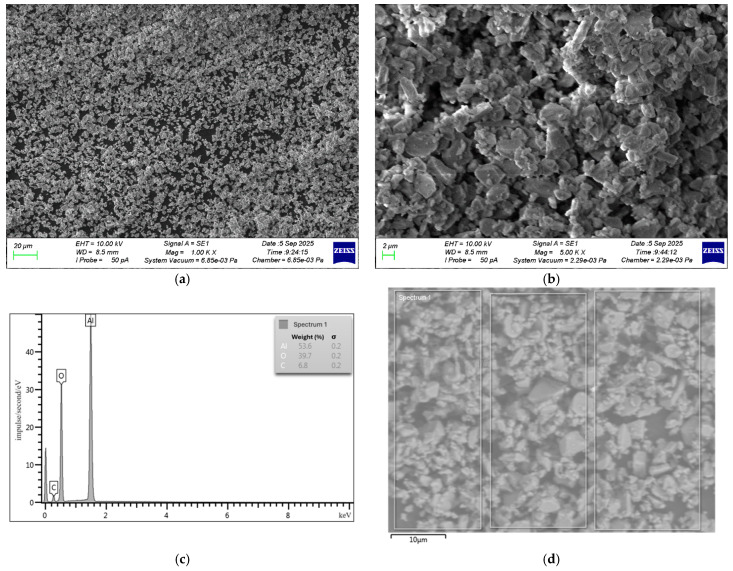
SEM and EDS results: (**a**) SEM image of NA at 1000× magnification; (**b**) SEM image of NA at 5000× magnification; (**c**) EDS spectrum; and (**d**) electronic image of the EDS scanning area.

Nanosized alumina (NA) particles (Shandong Tiancheng Chemical Co., Ltd., Yanzhou, China) were used as a nanomodifying additive. The SEM and EDS results for the NA particles are demonstrated in Figure 3.

NA particles show agglomeration, as clusters of smaller particles are visible on the surfaces of bigger ones. This is characterized by angular, plate-like particle shapes. EDS scanning was performed for the region (spectrum 1) shown in Figure 3d. EDS analysis identified Al, O, and C as the chemical elements present, with percentages of 53.55%, 39.69%, and 6.76%, respectively.

Rospena (Rospena, Moscow, Russia) was used as a foaming agent with density 1.1 g/cm^3^, and stability 1–3.5 h.

Polypropylene fiber (PF) (CEMMIX, Moscow, Russia) was used for dispersed reinforcement. PF characteristics were as follows: length—10–12 (mm); diameter—34 (μm); density—0.91 (g/cm^3^); tensile strength—320 MPa; and elastic modulus—6 GPa. The choice of PF with a fiber length of 10–12 mm was justified by the foam concrete mixture preparation technology. Based on preliminary laboratory studies, the optimal process parameters for foam concrete mixture preparation were determined, and the order of raw material addition during mixing was determined. Experiments showed that a fiber length of 10–12 mm is optimal given the existing process characteristics of the concrete mixing plant. PF fibers with a length of 10–12 mm are evenly distributed throughout the foam concrete mixture, minimizing the formation of PF agglomerates.

**Figure 4:**
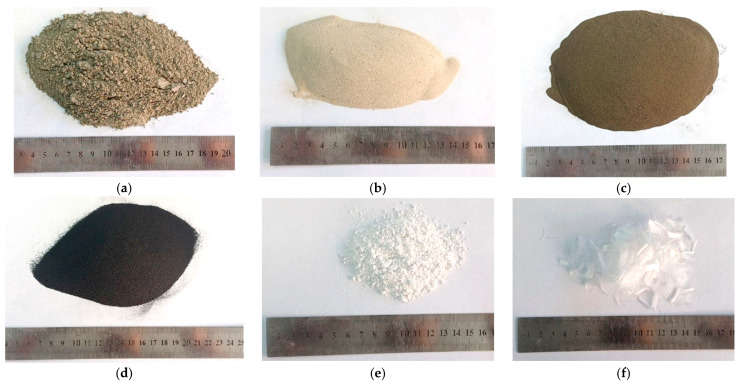
Appearance of raw materials: (**a**) PC; (**b**) QS; (**c**) FA; (**d**) MS; (**e**) NA; and (**f**) PF.

The primary raw materials’ appearance is illustrated in Figure 4.

**Figure 5:**
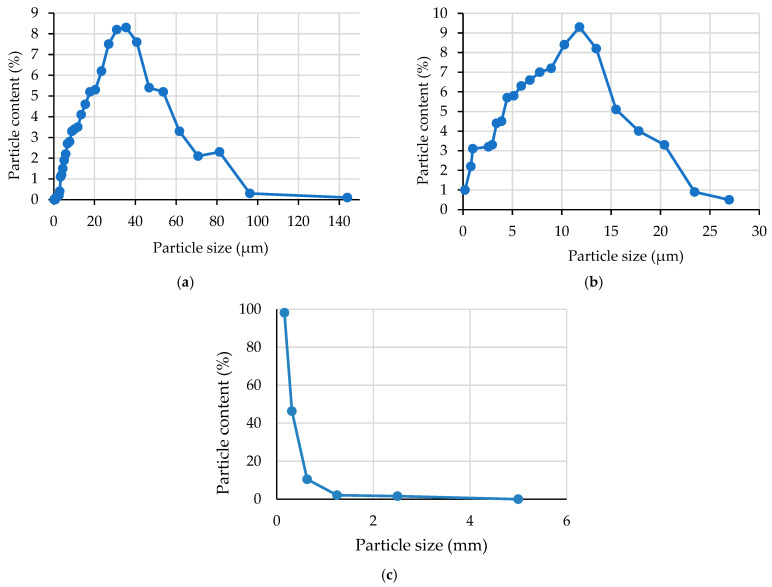
Particle size distribution curves: (**a**) FA; (**b**) MS; and (**c**) QS.

The particle size distributions of the raw materials are shown in Figure 5.

The particle size distribution curves in Figure 5 show the following:

– The majority of FA particles (81.1%) fall in the range of 8 to 62 µm;
– MS particles are up to 27 µm in size and are distributed as follows. A total of 33.2% of the particles are 5 µm or below, and 65.9% are 20 µm or below;
– The fineness modulus of QS is 1.59.

### 2.2. Methods

There were two central stages in this experimental investigation. The first involved the preparation of a complex nanomodifying additive (CNA) based on industrial waste and NA particles. Experimental foam concrete (FC) samples were created in the second stage, with different amounts of the complex nanomodifying additive and polypropylene fiber (PF). The optimal modification parameters for CNA and PF were determined.

*Stage 1. Method of manufacturing CNA.* First, FA was purified from impurities by sifting through “sieves with mesh sizes of 5 mm and 0.315 mm”. Then, the purified FA was dried in a laboratory drying oven to constant weight. To eliminate lumps, the MS was passed through a sieve with a 0.315 mm mesh. Constant weight was achieved by drying the sifted MS in a laboratory oven. Then, pre-cleaned and dried FA and MS were dosed in the required amount and poured into one container. In addition, NA additive and powdered plasticizing additive C-3 (CEMMIX, Moscow, Russia) were added to the FA and MS mixture. The plasticizing additive was added as a grinding intensifier. The mixture of mineral components for obtaining CNA was prepared in the following ratio: FA—80%; MS—18%; and NA—2%. Plasticizing additive C-3 was added at a rate of 0.2% of the final mass of the FA, MS, and NA mixture. The prepared mixture was mixed in an “Activator-4M planetary ball mill for 2 min at 600 rpm”. Mechanical activation ensures more uniform mixing of all components and further increases their specific surface area. The milling process also alters the particle surface structure, creating defects in the mineral lattices that accelerate elementary interactions of the particle surface layer, increasing their homogeneity and chemical activity [56,57,58].

*Stage 2. Fabrication of experimental FC and FFC samples.* Table 3’s recipe and the main process steps were observed to fabricate the FC modified with CNA. Initially, the necessary amounts of all raw materials were administered. Next, the components were thoroughly blended in this order: cement, sand, water, foaming agent, CNA, and PF. The completed mix was transferred to cube molds of 100 × 100 × 100 mm and prism molds measuring 100 × 100 × 400 mm. For the production of plate samples, the mixture was poured into cube molds to a depth of 2 cm. The FC samples were taken out of their molds 24 h post-production and then underwent 27 days of curing prior to testing. The samples underwent curing under conditions of 20 ± 2 °C temperature and 60 ± 10% relative humidity.

**Figure 6:**
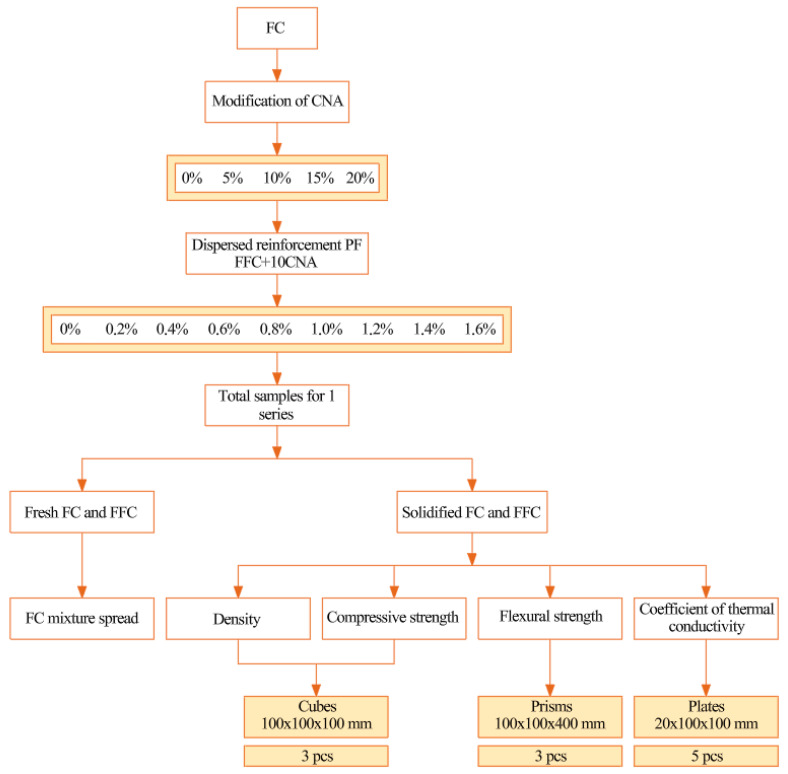
Experimental study scheme for FC-modified CNA and dispersion-reinforced PF.

Figure 6 presents the FC studies’ experimental design.

Overall, 39 cube specimens were used to measure density and compressive strength. Flexural strength was determined using 39 prism specimens. The thermal conductivity was assessed with the help of 65 plate specimens.

Standardized methods detailed in Table 4 were used to ascertain the workability of fresh FC and FFC, along with the primary physical and mechanical characteristics of hardened composites. In the laboratory room where the FC and FFC tests were performed, a temperature of (20 ± 5) °C and a relative humidity of 55–60% were maintained. All experimental specimens were visually inspected before testing. The presence of defects was recorded: cracks, rib chips, voids, and insufficient compaction of the structure. FC and FFC specimens with cracks and spalls greater than 10 mm deep, voids greater than 10 mm in diameter, and traces of insufficient compaction of the foam concrete mixture were not allowed to undergo testing [59,60,61,62,63,64,65,66].

XRD analysis was performed on a DRON-7 diffractometer (Burevestnik, St. Petersburg, Russia) using copper radiation (wavelength 1.54 Å) with good resolution (0.025 step, 1 s exposure).

A ZEISS CrossBeam 340 (Carl Zeiss Microscopy GmbH, Jena, Germany), a dual-beam scanning electron ion microscope with an Oxford Instruments X-Max 80 X-ray microanalyzer (Oxford Instruments, Abingdon, UK) was used to examine the particle surface morphology and elemental composition of FA, MS, and NA, along with the microstructure of the composites.

## 3. Results and Discussion

### 3.1. Results of CNA Property Evaluation

**Figure 7:**
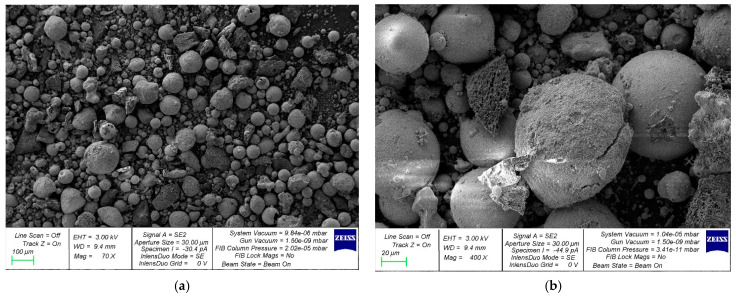
SEM image of CNA: (**a**) at 70× magnification; and (**b**) at 400× magnification.

**Figure 8:**
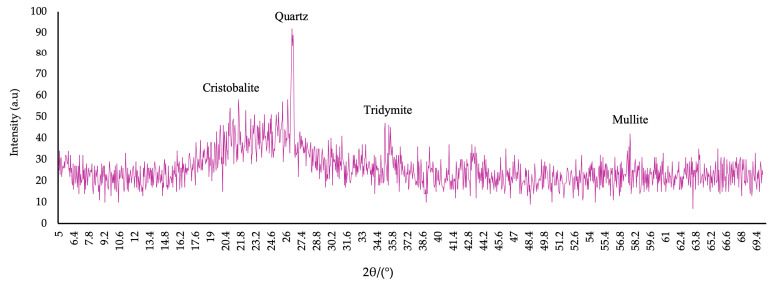
CNA X-ray diffraction pattern.

The SEM and XRD analyzes results of CNA are presented in Figure 7 and Figure 8, respectively.

CNA is a mixture of FA and MS particles and NA nanoparticles. The MS and NA particles are uniformly distributed among the spherical FA particles. No agglomerated particle clusters were observed.

The CNA X-ray diffraction pattern predominantly shows the amorphous phase. Reflections of mullite, quartz, tridymite, and cristobalite are recorded.

### 3.2. Results of Evaluating the Properties of CNA-Modified FC

**Figure 9:**
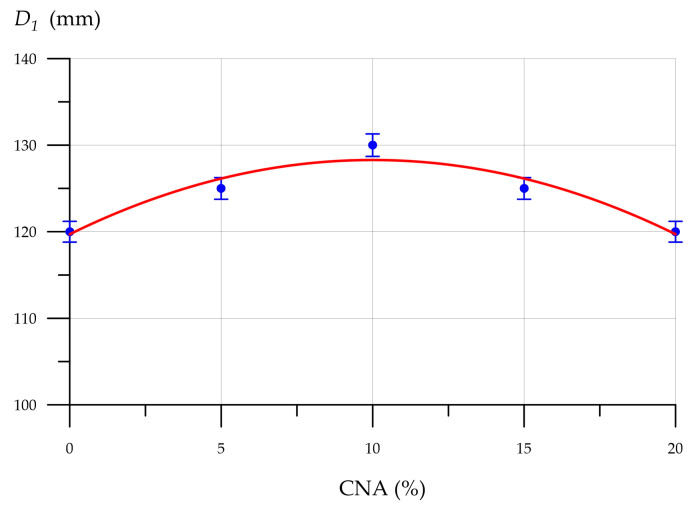
FC mix flow diameter (*D*_1_) vs. CNA content.

**Figure 10:**
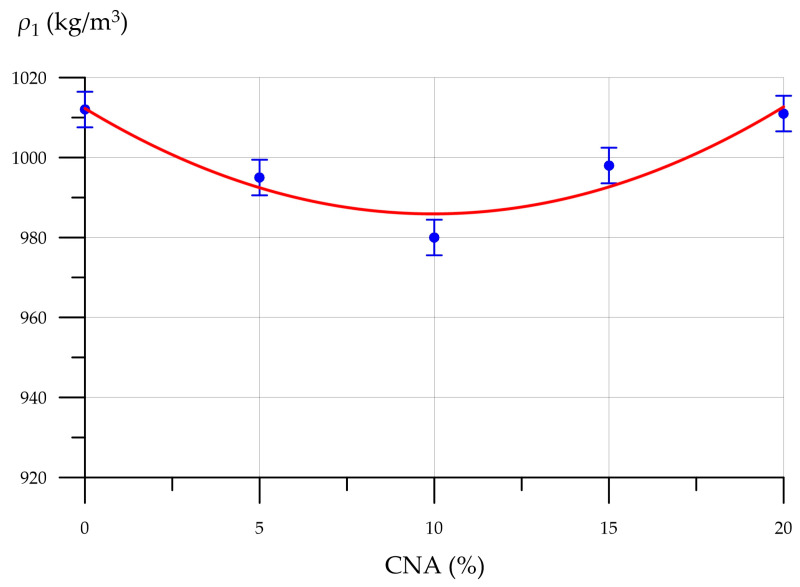
Dependence of FC density (*ρ*_1_) on CNA content.

**Figure 11:**
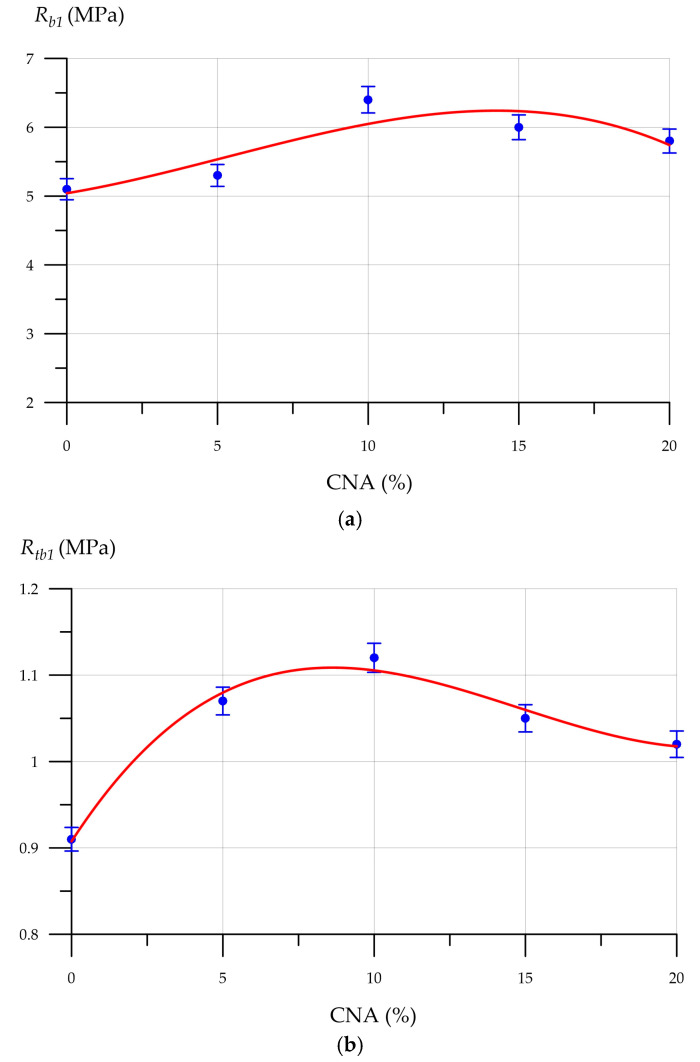
FC strength properties as a function of CNA content: (**a**) compressive strength (*R_b_*_1_); and (**b**) flexural strength (*R_tb_*_1_).

**Figure 12:**
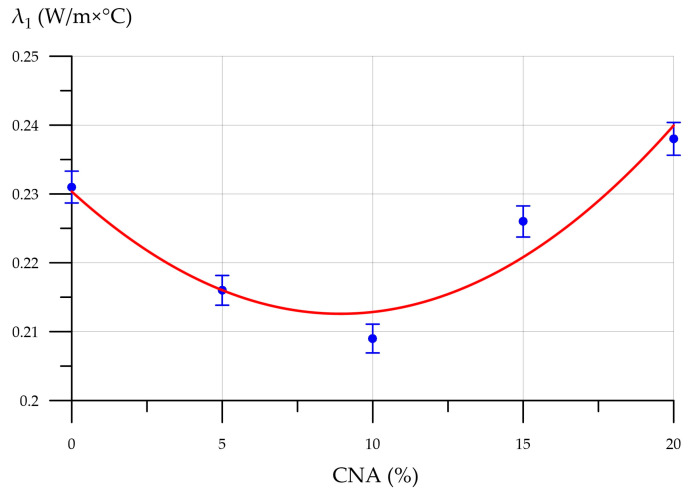
Thermal conductivity of FC (*λ*_1_) versus CNA content.

Figure 9, Figure 10, Figure 11 and Figure 12 show the mixture flow rate, density, compressive strength, flexural strength, and thermal conductivity of CNA-modified FC. Figure 9 illustrates how the FC mixture flow rate diameter changes with CNA content.

As Figure 9 demonstrates, the flow diameter of FC mixtures modified with CNA increases to 130 mm with the addition of 10% CNA. Subsequently, as the CNA dosage increased, a slight decrease was observed, and the melt diameter of the mixture with 20% CNA content was 120 mm, which is comparable to the control value. The observed increases in the FC mixture flow diameter when replacing part of the cement with 10% CNA are due to the presence of NA nanoparticles and an additional powdered plasticizer additive in the CNA composition. Figure 10 shows the dependence of FC density on CNA content.

This demonstrates the relationship between the FC density change and CNA content. The introduction of CNA up to and including 10% reduces the composite density to a minimum of 980 kg/m^3^. Then, with increasing CNA dosage, the density increases again, reaching 1011 kg/m^3^ at 20%. The lowest FC density at a 10% CNA content is explained by the increased packing efficiency and uniformity of the void distribution within the foam concrete matrix. Through these mechanisms, the mineral components in CNA improve foam stability and stop bubbles from collapsing. FA particles cling to foam bubbles, impeding the dispersion medium’s downward movement due to gravity, which, consequently, enhances the foam concrete mixture’s stability. MS particles on the surface of foam bubbles form channels through which the inter-bubble diffusion of gas occurs, slowing the process of foam bubble coarsening and increasing sedimentation stability. NA are adsorbed at the gas–liquid interface and form a strong bond with the liquid film, while NA particles distributed between the bubbles create capillary pressure, which prevents their merging. Thus, due to the increased foam stability due to the CNA modification, the total number of pores in the FC structure increases, and the density of the composite modified with the optimal amount of CNA decreases. However, at higher CNA dosages (more than 10%), this stabilizing effect decreases [67,68]. The relationship between compressive strength, flexural strength, and CNA content is displayed in Figure 11.

The equations describing the change in the compressive strength and flexural strength of FC with different CNA dosages are represented by the following functions:

(1) $$R_{b1}=-0.0005x^{3}+0.0074x^{2}+0.0731x+5.0414\;\;R_{2}=0.7833$$

(2) $$R_{tb1}=0.0001x^{3}-0.0044x^{2}+0.0541x+0.9076\;\;R_{2}=0.9832$$

The dependences of FC strength properties on CNA content, shown in Figure 11, are similar. CNA modification improves the strength properties of FC in all studied ranges up to and including 20%. With 10% CNA, the FC composite achieved a maximum compressive strength of 6.4 MPa and flexural strength of 1.12 MPa, exceeding the control by 25.5% and 23.1%. The CNA modification’s effectiveness declines when applied at 15% and 20%. Therefore, FC containing 20% CNA exhibits 5.8 MPa compressive strength and 1.02 MPa flexural strength, surpassing the control values by 13.7% and 12.1%, respectively. All mineral elements within the composite contribute to the enhanced strength observed after CNA modification. The overall mechanism of CNA operation can be described as follows. Mechanically activated FA and MS particles are involved in hydration reactions, converting Ca(OH)_2_ into a hydrosilicate gel. The newly created silicate gel blocks the capillary pores, making the cement matrix denser. The composite material’s overall strength is enhanced because hydrosilicate gels reinforce the interpore partition structure [69,70]. The relationship between FC thermal conductivity and CNA content is illustrated in Figure 12.

The inclusion of CNA in FC improves its thermal insulation properties. Thermal conductivity decreases with CNA content up to and including 10%. With 10% CNA, the lowest thermal conductivity was measured at 0.209 W/m×°C, which is 9.5% less than the control. As the CNA content rises to 20%, an inverse relationship is noted. The FCs with the highest CNA content (20%) exhibit a thermal conductivity of 0.238 W/m×°C, a 3% increase over the control. The decrease in the thermal conductivity of cellular composites modified with a complex nanomodifying additive based on industrial waste up to 10% is due to its stabilizing properties and high pozzolanic activity. Modified FCs have a more homogeneous structure with uniformly distributed pores. Moreover, due to the presence of additionally formed CSH gels, the density of interpore partitions increases, improving the thermal insulation properties of the entire composite [71,72]. However, at higher CNA contents, this effect is reduced because the FC mixture is supersaturated with FA particles, leading to the merging and collapse of bubbles. The composite’s thermal conductivity rises because the pore count drops while dense areas multiply. Table 5 illustrates the changes in the properties of FC that has been modified with CNA.

Experimental studies have shown that the inclusion of CNA in FC is a rational formulation solution and significantly improves the strength properties of the composite. By substituting up to 20% of Portland cement with CNA, FC’s strength has improved. Environmentally friendly structural and thermal insulation foam concrete, with enhanced properties, can be produced using the CNA additive, which is derived from industrial waste. However, the 10CNA composition exhibits the best strength properties combined with the lowest density and thermal conductivity. Therefore, the 10CNA composition is used as the basis for further experimental studies to select the optimal reinforcement parameters for PF.

### 3.3. Results of Evaluating the Properties of FFC with PF Modified with CNA

**Figure 13:**
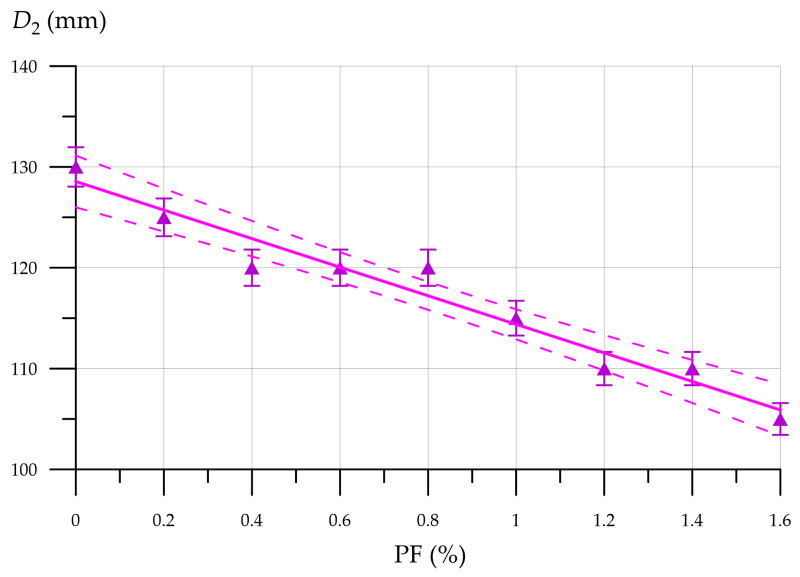
FFC mix flow diameter vs. PF content.

**Figure 14:**
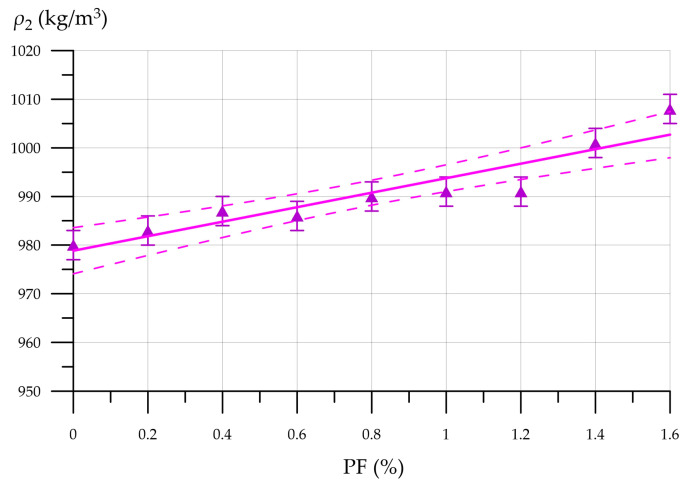
FFC density with CNA versus PF content.

**Figure 15:**
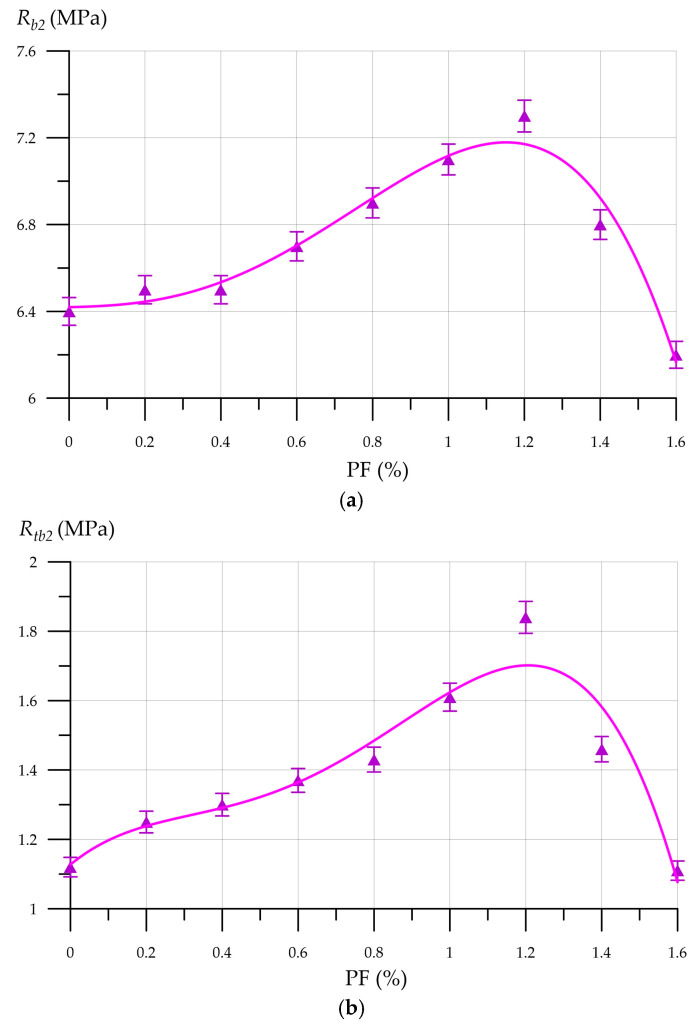
FFC with CNA properties as a function of PF content: (**a**) compressive strength; and (**b**) flexural strength.

**Figure 16:**
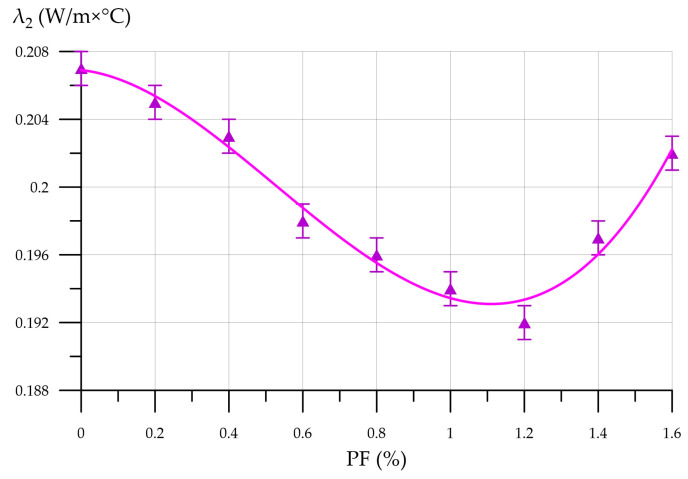
Thermal conductivity of FFC with CNA versus PF content.

Figure 13, Figure 14, Figure 15 and Figure 16 showcase the determined mixture flow, density, compressive strength, flexural strength, and thermal conductivity of FFC modified with PF and CNA. The spread diameter of the FFC mixture, as depicted in Figure 13, is dependent on the CNA content.

The flow diameter of foam concrete mixtures modified with 10% CNA and reinforced with PF tends to decrease as the PF amount increases from 0.2% to 1.6%, inclusive. The flow diameter of the FFC mixture with the maximum 1.6% PF content was 105 mm, which is 19.2% less than the flow diameter of the 10CNA mixture. The reduced flow of fiber-reinforced foam concrete is because PF mixes with the mortar, reducing the flow rate of the mixture as the cylinder rises. This phenomenon intensifies with an increasing PF content in the FC. The relationship between FFC density, CNA, and PF content is depicted in Figure 14.

Figure 14 shows that the FFC density increases with increasing PF dosage, ranging from 980 kg/m^3^ to 1008 kg/m^3^. Figure 15 shows the compressive and flexural strength of FFC with CNA versus PF content.

The equations describing the change in compressive strength and flexural strength of FFC with CNA and different PF dosages are represented by the following functions:

(3) $$R_{b2}=-1.9886x^{3}+3.7689x^{2}-1.0985x+6.4636\;\;R_{2}=0.9413$$

(4) $$R_{tb2}=-1.1353x^{3}+2.1033x^{2}-0.4904x+1.181\;\;R_{2}=0.8443$$

Figure 15a illustrates the relationship between the compressive strength of FFC with the CNA and PF content, revealing a specific trend. The highest compressive strength of 7.3 MPa, a 43.1% improvement over the control, is found at 1.2% PF, following an increase between 0.2% and 1.2%. Moreover, once the PF content exceeds 1.2%, dispersed reinforcement becomes less effective. The compressive strengths for composites with 1.4% and 1.6% PF are 6.8 MPa and 6.2 MPa. The flexural strength curve has a similar pattern, with the maximum value of 1.84 MPa observed for the composition with 1.2% PF. The foam concrete matrix is stabilized by PF fibers, which control the solid-phase movement during hydration and cement matrix development. Due to the uniform distribution of PF in the foam concrete matrix, dense structural clusters are formed, which create favorable conditions for late pozzolanic reactions. The presence of such structural clusters forms dense bonds at the phase boundaries, which subsequently increases the strength properties of FFC. The effectiveness of PF reinforcement decreases when the number of fibers exceeds the optimal value [73]. PF fibers begin to tangle and form lumps. Unevenly distributed PF fibers and lumps of entangled fibers deteriorate the structure of the entire composite, forming weak zones in it and reducing the strength properties. The relationship between the PF content and the thermal conductivity of FFC with CNA is depicted in Figure 16.

The thermal conductivity of FFC with CNA reinforced with PF tends to decrease with dispersed reinforcement parameters ranging from 0.2% to 1.2%, respectively. The minimum thermal conductivity of 0.192 W/m×°C was recorded for the 10CNA1.2PF composition, which is 16.9% lower than the control value. Starting with 1.4% PF, the thermal conductivity increases, reaching 0.197 W/m×°C and 0.202 W/m×°C for the 10CNA1.4PF and 10CNA1.6PF compositions, respectively. The introduction of polypropylene fibers at an optimal dosage of up to 1.2% stabilizes the structure of the foam concrete matrix. PF fibers are distributed throughout the composite, forming a spatial framework that reduces heat flow and improves its thermal insulation properties [74,75,76,77].

### 3.4. Results of SEM Analysis of FC and FFC Structures

**Figure 17:**
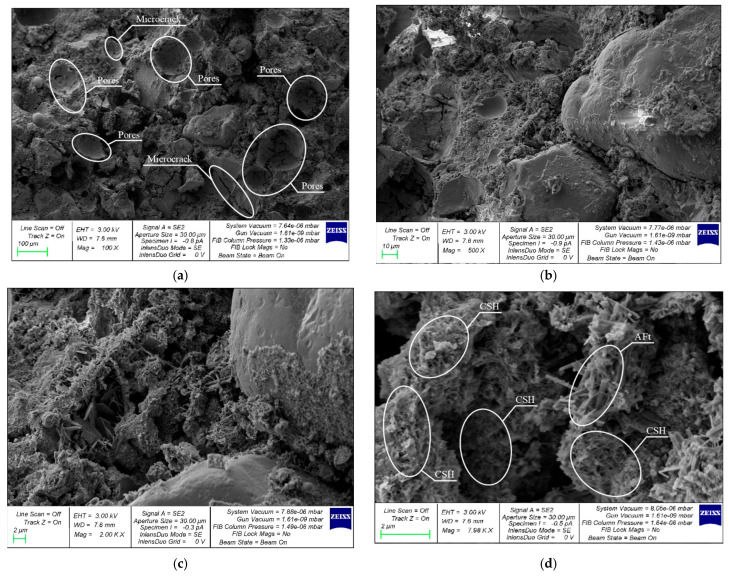
SEM analysis of the structure of the 0CNA0PF control composition with magnifications of (**a**) 100×; (**b**) 500×; (**c**) 2000×; and (**d**) 7980×.

**Figure 18:**
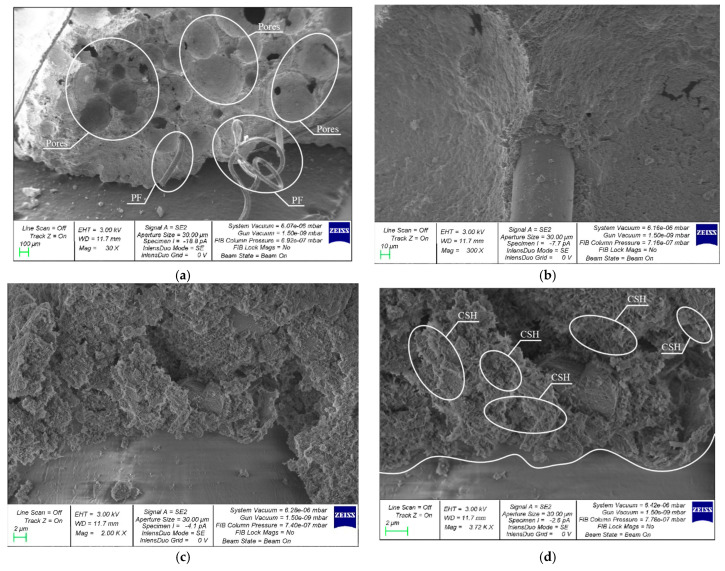
SEM analysis of the structure of the 10CNA1.2PF composite: (**a**) 100×; (**b**) 500×; (**c**) 2000× and (**d**) 80,000×.

A comparative analysis of the microstructure of the FC control composition and FFC with 1.2% PF modified with 10% CNA was performed. Figure 17 and Figure 18 show morphological images of the microstructure of foam concrete (Figure 17) and fiber-reinforced foam concrete (Figure 18) at various magnifications.

The foam concrete matrix’s pore structure can be seen in Figure 17. The pore structure of foam concrete consists of closed pores in the form of round cells, interpore partitions, and clusters of newly formed crystalline formations. At the interface between the cement matrix and the aggregate grain, the contact zone is loose. Multiple microcracks and voids are observed on the inner surfaces of the pore cell walls. The structure of the interpore partitions is disorganized, with microcracks and pores also observed. At higher magnifications, clusters of CSH and ettringite (Aft) zones are observed in zones on the inner surfaces of the pore cell walls.

The pore structure of the fiber-reinforced foam concrete matrix is represented by closed pores in the form of round cells, interpore partitions, clusters of crystalline formations and PF fibers. The structure of the fiber-reinforced foam concrete matrix, shown in Figure 18, is more organized and homogeneous compared to the structure of the foam concrete matrix of the control composite. The pore cells are uniformly distributed. PF fibers are predominantly located in the interpore partition zones. Microcracks are observed in smaller numbers on the inner surfaces of the pore cell walls. The zone at the cement matrix–PF fiber interface is dense, with multiple clusters of CSH zones. The tight adhesion of the cement matrix to the PF fiber and the presence of CSN indicate the high adhesion of the fiber to the cement matrix.

The experimental studies revealed that the modification of CNA and PF foam concretes allows for the production of environmentally friendly composites with improved performance properties. During the process of determining the optimal amount of CNA, it was established that the best physico-mechanical properties were showed by a 10CNA0PF composition containing 10% of a complex nanomodifying additive based on industrial waste with the addition of NA nanoparticles and a powdered plasticizer. During the determination of the optimal parameters for dispersed reinforcement, the highest increases in strength properties were demonstrated with the inclusion of 1.2% PF. An indirect indicator for assessing the effectiveness of the applied formulation techniques is the structural quality coefficient (CCQ), which is determined by the ratio of changes in the strength and density of the composite and is calculated using the following formula:

(5) $$CCQ=\frac{R}{\rho }$$

where:

*R* is the compressive strength (MPa);

*ρ* is the density (g/cm^3^).

**Figure 19:**
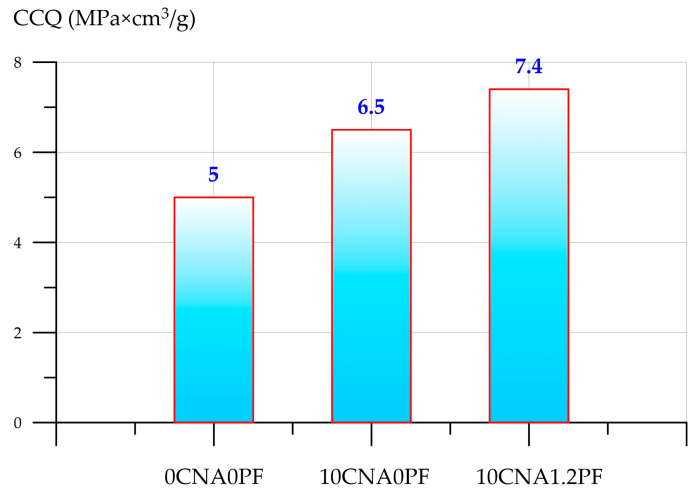
CCQ determination results.

The results of the CCQ calculations for the control, 10CNA0PF, and 10CNA1.2PF foam concrete composites are shown in Figure 19.

The structural quality coefficients are higher for FC and FFC compositions modified with CNA when contrasted with the control composition. The CCQ of foam concrete with 10% CNA increased by 30%, while, for fiber-reinforced foam concrete, it increased by 48%, respectively.

In this study, the tested formulation techniques lead to better physical and mechanical characteristics for FC and FFC, which corresponds with the results of other authors (Table 6).

The study revealed that, for mineral additives like fly ash and microsilica, the ideal proportion in foam concrete is 5–30%. For PF reinforcement, the optimal range is 0.1–1.5%, which is consistent with previous research. The improved strength and thermal insulation properties of FC and FFC are explained by the following key mechanisms:

– The main components of CNA are FA and MS, the chemical composition of which is primarily represented by the oxides SiO_2_, Al_2_O_3_, and Fe_2_O_3_. These components have high pozzolanic activity and actively interact with Ca(OH)_2_, resulting in the formation of additional hydrosilicate gels. The presence of additional CSH strengthens the structure of the interpore partitions, thereby increasing the overall strength of foam concrete;
– NA nanoparticles act as a stabilizer, preventing the merging of foam bubbles and promoting the formation of a more uniform cellular structure; the presence of a uniform pore structure and reinforced interpore partitions improves the thermal insulation properties of foam concrete;
– The presence of a powdered plasticizer in the CNA composition compensates for the increased water demand of finely dispersed mineral components and promotes the better distribution of FA, MS, and NA particles within the foam concrete matrix;
– PF fibers are uniformly distributed throughout the foam concrete matrix, intertwining to create a spatial reinforcing framework; the presence of a stable fiber framework allows for stress redistribution and prevents microcrack formation;
– A complex nanomodifying additive combined with polypropylene fibers influences the failure mode of the composite. Under mechanical loads, including bending, the composite exhibits a viscoplastic failure mode. Under load, the PF fibers are pulled out of the composite, and failure under critical loads is accompanied by fiber rupture at the cement matrix–PF fiber interface. Nanoparticles increase the strength of interpore walls, making them more resistant to destructive loads.

The results obtained in this study are of significant importance for the construction industry. A complex modifying additive based on industrial waste and nanomaterials was developed, and the optimal ratio of fly ash, microsilica, and aluminum oxide nanoparticles in the CNA composition was selected. A method for the preparation and mechanical activation of the main components of CNA is described. The mechanical activation of the CNA components destroys and transforms the microstructure of FA and MS particles, thereby releasing reactive components and increasing the overall pozzolanic activity of the complex additive [87]. The finished additive, based on industrial waste and nanomaterials, has great commercial potential. FC and FRFC produced using CNA and PF have improved durability and thermal insulation properties. Using industrial waste to create CNA, which then replaces some cement in foam concrete composites, benefits the environment and promotes sustainability. The study focuses on identifying the key physical and mechanical characteristics of foam concrete using CNA. Future research aims to assess the durability of FC and FFC, including their water absorption, frost resistance, and chemical resistance. Comparative studies will also be conducted on the microstructural changes in the composites after exposure to aggressive environments.

## 4. Conclusions

In this study, energy-efficient and environmentally friendly foam concretes modified with a complex nanomodifying additive based on industrial waste and aluminum oxide nanoparticles and reinforced with polypropylene fiber were developed. The properties of foam concrete and fiber-reinforced foam concrete were assessed for density, compressive and flexural strength, and thermal conductivity. The microstructural features of the manufactured composites were studied, and the structural quality factors for the best compositions were calculated;

(1) A complex nanomodifying additive based on industrial waste was developed with the following composition: FA—80%; MS—18%; NA—2%; and C-3 plasticizing additive—0.2%. The X-ray diffraction pattern of CNA shows a predominantly amorphous phase, demonstrating its high activity.
(2) The inclusion of CNA at levels up to 10% reduces the density of the cellular composites. The lowest density value of 980 kg/m^3^ was recorded for FC with 10% CNA. The density of FFC varied from 983 kg/m^3^ to 1008 kg/m^3^.
(3) The inclusion of up to 20% CNA in place of part of the cement has a positive effect on the strength properties of foam concrete. CNA demonstrates the greatest effectiveness at a level of 10%. The increases in compressive and flexural strength were 25.5% and 23.1%, respectively. Dispersed PF reinforcement in combination with CNA enhances the effect and further improves strength properties. The optimal percentage of dispersed PF reinforcement is 1.2% and, together with CNA, provides an increase in compressive strength of up to 43.1% and flexural strength of up to 102.2% compared to control samples.
(4) The thermal insulation properties of FC are improved with the inclusion of CNA. The FC composition with 10% CNA exhibited the lowest thermal conductivity coefficient of 0.202 W/m×°C, which is 9.5% lower than that of the control composition. The addition of PF enhances the effect of CNA modification. For FFC with 1.2% PF, the thermal conductivity coefficient was 0.192 W/m×°C, which is 16.9% lower than the control.
(5) A microstructural analysis of the foam concrete composite modified with 10% CNA and reinforced with 1.2% PF confirms the effectiveness of the formulation techniques. The cellular structure of the modified composite is uniform. The PF fibers adhere fairly tightly to the cement matrix. Multiple clusters of CSH zones are observed at the phase boundary. The increases in the structural quality factors for the composition with 10% CNA and with 10% CNA + 1.2PF were 30% and 48%, respectively.
(6) The foam concrete composites obtained during the study are classified as structural and thermal insulation materials and can be used in civil and industrial construction.

## Figures and Tables

**Table 1:** PC properties.

Indicator Name		Actual Value	
Specific Surface Area (m^2^/kg)		350	
Normal Density (%)		29	
Start of Setting (hours—min)		3–10	
End of Setting (hours—min)		4–40	
Compressive Strength after 28 Days (MPa)		52.3	
Bending Strength after 28 Days (MPa)		8.1	
Uniform Volume Change (mm)		1.1	
**Mineralogical composition**			
C_3_S (%)	C_2_S	C_3_A	C_4_AF
67.5	12.9	7.1	12.5

**Table 2:** QS properties.

Indicator Name	Actual Value
Bulk density (kg/m^3^)	1348
Apparent density (kg/m^3^)	2575
The content of dust and clay particles (%)	0.12

**Table 3:** FC and FFC compositions with flow rates per 1 m^3^.

Num.	Mixture Type	PC (kg/m^3^)	QS (kg/m^3^)	CNA (kg/m^3^)	PF (kg/m^3^)	W (L)	Rospena (kg/m^3^)
1	0CNA0PF	325.0	530	0	0	160	9.75
2	5CNA0PF	308.8	530	16.2	0	160	9.75
3	10CNA0PF	29.5	530	32.5	0	160	9.75
4	15CNA0PF	276.3	530	48.7	0	160	9.75
5	20CNA0PF	260.0	530	65	0	160	9.75
6	10CNA0.2PF	292.5	530	32.5	0.65	160	9.75
7	10CNA0.4PF	292.5	530	32.5	1.3	160	9.75
8	10CNA0.6PF	292.5	530	32.5	1.95	160	9.75
9	10CNA0.8PF	292.5	530	32.5	2.6	160	9.75
10	10CNA1.0PF	292.5	530	32.5	3.25	160	9.75
11	10CNA1.2PF	292.5	530	32.5	3.9	160	9.75
12	10CNA1.4PF	292.5	530	32.5	4.55	160	9.75
13	10CNA1.6PF	292.5	530	32.5	5.2	160	9.75

**Table 4:** FC and FFC property testing methods.

Property Name	Test Parameters	Formula
Suttard Spread Diameter (D, mm) [59]	“The Suttard viscometer and glass plate (Promkomplekt, Rostov-on-Don, Russia) were first wiped with a damp cloth. The viscometer was then centered on the plate and filled with the mixture, with any excess being trimmed off with a ruler. The viscometer was then raised to a height of 20 cm. The diameter of the spread was measured immediately after lifting the cylinder with a ruler in two perpendicular directions with an error of no more than 5 mm, and the arithmetic mean was calculated” [59].	The spread diameter of the FC and FFC mixture according to Suttard was calculated using the formula: $D=\frac{D_{1}-D_{2}}{2}$ *D*_1_ and *D*_2_—spread diameters in two perpendicular directions, mm
FC and FFC Density (ρ, kg/m^3^) [60]	The FC and FFC samples were dried to constant weight, their geometric parameters were measured, and weighed.	The density of FC and FFC was calculated with an accuracy of 1 kg/m^3^ using the formula: $\rho =\frac{m}{V}\cdot 1000$ where m is the sample mass (g) and V is the sample volume (cm^3^). In each series of samples, the density was determined as the arithmetic mean of the test results of three samples.
FC and FFC Compressive Strength (*R_b_*, MPa) [61,62,63,64]	Before determining the compressive strength, the FC and FFC samples were dried to constant weight, then placed in the testing machine (ZIM, Armavir, Russia) and forced to collapse at a constant rate of (0.6 ± 0.2) MPa/s.	The compressive strength of FC and FFC was calculated with an accuracy of 0.1 MPa using the formula: $R=\alpha \frac{F}{A}K_{w}$ where *F* is the failure load (N); *A* is the cross-sectional area of the specimen (mm^2^); α is a coefficient accounting for the specimen dimensions (for specimens with a side length of 100 mm, α = 0.95); and *Kw* = 0.8 “The compressive strength of a series of specimens was calculated as the arithmetic mean of the two specimens with the highest strength out of three”.
FC and FFC Flexural Strength (*R_tb_*, MPa) [65]	Before determining the flexural strength, FC and FFC specimens were dried to constant weight, then mounted in a testing machine (ZIM, Armavir, Russia) and loaded to collapse at a constant rate of (0.05 ± 0.01) MPa/s.	The flexural strength of FC and FFC was calculated with an accuracy of 0.1 MPa using the formula: $R=\delta \frac{Fl}{ab^{2}}K_{w}$ where *F* is the failure load (N); *a*, *b*, and *l* are the width and height of the prism cross-section, and the distance between supports (mm); δ = 0.92; and *Kw* = 0.8. “The flexural strength of a series of samples was calculated as the arithmetic mean of three samples, based on the two with the highest strength”.
FC and FFC Thermal Conductivity (λ, W/m×°C) [66]	The thermal conductivity of the experimental FC and FFC specimens, dried to constant weight, was assessed under steady-state thermal conditions. Before testing, the plate edges were ground and their thickness measured. The specimens were then placed in an ITP-MG4 thermal conductivity tester (SKB Stroybribor, Chelyabinsk, Russia) and the measurements were performed.	“The thermal conductivity coefficient of a series of samples was determined as the arithmetic mean of the test results of five samples”.

**Table 5:** Changes in the properties of FC modified with CNA.

Change of Properties	CNA (%)				
	0	5	10	15	20
∆D_1_ (%)	0	4.2	8.3	4.2	0
∆$\rho_{1}$(%)	0	−1.7	−3.2	−1.4	−0.1
∆R_b1_ (%)	0	3.9	25.5	17.6	13.7
∆R_tb1_(%)	0	17.6	23.1	15.4	12.1
∆λ_1_ (%)	0	−6.5	−9.5	−2.2	3.0

**Table 6:** Effect of formulation techniques on the properties of foam concrete.

Ref. Num.	Additive Type	Optimal Dosage	Result Obtained
[78]	FA + quick-hardening cement	Up to 30%	Improved thermal insulation properties of the composite.
[79]	FA	Up to 5%	Decreased density and increased compressive strength.
[80]	A mixture of mineral additives based on FA, MS, and metakaolin + PF	10%/0.8	Improved pore structure and increased strength properties.
[81]	An additive based on FA and rubber powder	Up to 10%	Increased compressive and flexural strength.
[82]	SiO_2_/PF	Up to 5%/0.1	“Strength increased by 16.67% and thermal conductivity decreased by 12.5%”. [82]
[47]	PF	1.5%	Bending strength increased by 84%.
[83,84,85]	PF	From 0.6% to 1%	Polypropylene fiber acts as a binder and improves composite integrity. Water absorption is reduced, improving performance properties.
[86]	NA	Up to 2%	Foam concrete samples demonstrate increased strength and frost resistance.
[30]	NA	0.18%	Nanoparticles prevent bubble aggregation and improve foam stability.

## Data Availability

The original contributions presented in this study are included in the article. Further inquiries can be directed to the corresponding author.

## Abbreviations

The following abbreviations are used in this manuscript:

PF	Polypropylene fiber
CNA	Complex nanomodifying additive
FC	Foam concrete
FFC	Fiber-reinforced foam concrete
FA	Fly ash
CCQ	Structural quality coefficient
